# Risk for cardiovascular disease in Japanese patients with rheumatoid arthritis: a large-scale epidemiological study using a healthcare database

**DOI:** 10.1186/s40064-016-2800-6

**Published:** 2016-07-19

**Authors:** Kunihiko Tanaka, Kenji Hamada, Terumi Nakayama, Shinichi Matsuda, Akihide Atsumi, Tomomi Shimura, Masatomi Nemoto

**Affiliations:** Drug Safety Division, Chugai Pharmaceutical Co. Ltd., 1-1 Nihonbashi-Muromachi 2-Chome, Chuo-ku, Tokyo, 103-8324 Japan; Clinical Development Division, Chugai Pharmaceutical Co. Ltd., 1-1 Nihonbashi-Muromachi 2-Chome, Chuo-ku, Tokyo, 103-8324 Japan

**Keywords:** Rheumatoid arthritis, Cardiovascular disease, Japanese, Epidemiology, Healthcare database

## Abstract

**Objectives:**

To study risk for cardiovascular disease (CVD) in Japanese patients with rheumatoid arthritis (RA).

**Methods:**

We used a Medical Data Vision database mainly composed of health insurance claim data and diagnosis-procedure combination data from Japan. Patients with RA diagnosed from April 2011 to March 2014 at 71 hospitals were identified with the International Classification of Diseases 10th revision (ICD-10) and history of anti-RA drug prescription. Hospitalizations for CVD including ischemic heart disease, heart failure, and stroke were identified by a combination of diagnosis (ICD-10) and diagnostic procedures. CVD incidence rate ratio (IRR) for RA versus osteoarthritis was calculated. Risk factors were analyzed using univariate and multivariate Cox proportional hazard models with baseline C-reactive protein (CRP) and traditional risk factors as covariates.

**Results:**

We identified 8658 patients with RA. The age–sex adjusted IRR for RA versus osteoarthritis was high for total CVD [2.12; 95 % confidence interval (CI) 1.93–2.32], ischemic heart disease (2.16; 95 % CI 1.86–2.50), heart failure (2.34; 95 % CI 2.07–2.65), and stroke (1.68; 95 % CI 1.41–2.00). Risk factor analysis showed a tendency for cardiovascular risk to increase with higher baseline CRP, although the difference was not statistically significant (hazard ratio 1.43; 95 % CI 0.99–2.07).

**Conclusion:**

Our study indicates an increased risk for CVD and an association between systemic inflammation and CVD in Japanese RA patients.

## Background

Rheumatoid arthritis (RA) is a chronic disease with various complications and a higher mortality than that expected in the general population (Pincus et al. 1994). Cardiovascular disease (CVD) is one of the serious complications in RA and is a major cause of death in RA patients in both the West and Japan (Sihvonen et al. 2004; Nakajima et al. 2010). Non-clinical research has suggested that proinflammatory cytokines, which are present at higher levels in RA patients, play a role in the development of CVD (Libby 2008). Moreover, a correlation between increased levels of inflammatory marker C-reactive protein (CRP) and higher incidence of CVD has been observed in an epidemiological study (Goodson et al. 2005).

A higher CVD risk in RA patients has been observed in some epidemiological studies in Western countries (Maradit-Kremers et al. 2005; Avina-Zubieta et al. 2008; Peters et al. 2009). Although there is a known ethnic difference in CVD incidence in the general population (Menotti et al. 1993), few studies have explored CVD in RA patients in Japan. Recently, Sakai et al. (2016) reported high prevalence of cardiovascular comorbidities in patients with RA, using a database of medical claims from employment-based health insurance organizations in Japan. We used another healthcare database of diagnosis-procedure combination data, health insurance claims data, and laboratory data from hospitals located throughout Japan to study the relative risk of CVD as well as the association between systemic inflammation and CVD in Japanese patients with RA. Patients with osteoarthritis (OA) were used for comparison because inflammation is mostly confined to the affected joints in OA but is systemic in RA. OA also shares with RA both traditional and lifestyle risk factors for CVD (Pelletier et al. 2001).

## Methods

### Data source

The healthcare database used for this study was provided by Medical Data Vision Co., Ltd. The database contains Japanese diagnosis-procedure combination data and health insurance claims data from hospitals located throughout Japan, anonymously managed to protect patient’s personal information; some hospitals also provide laboratory data. This study used clinical data from 71 hospitals collected from April 2011 to March 2014, including laboratory data from 14 hospitals.

### Patients

**Fig. 1:**
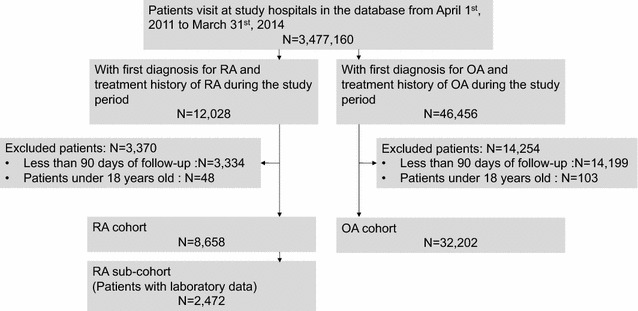
Flow chart of the study cohort. *RA* rheumatoid arthritis, *OA* osteoarthritis

Data were extracted for patients aged 18 years or older with RA or OA diagnosed for the first time during the study period, from April 2011 to March 2014, and with data available for a follow-up period of at least 90 days (Fig. 1). The following codes from International Classification of Diseases 10th revision (ICD-10) were used to identify cases for extraction: M05, M06.0, M06.2, M06.3, M06.8, and M06.9 for RA, and M179 for OA. Each patient was prescribed or had a treatment history of anti-RA drugs (DMARDs, oral corticosteroids, biologics, or oral NSAIDs) or OA treatments (oral or topical NSAIDs, hyaluronic acid injections, or corticosteroids injections) and was followed up until the end of March 2014 or until leaving the database. RA and OA patients in the sub-cohort were defined as patients with laboratory data for risk factor analyses.

### Outcomes

Outcomes are defined as events that occurred after first diagnosis of RA or OA and were identified by a combination of diagnoses (ICD-10) leading to hospitalization and medical tests. Diagnoses of CVD in this study were defined as ischemic heart disease [angina pectoris (I20) or myocardial infarction (I21, I22)], heart failure (I50), and stroke [cerebral infarction (I63), intracerebral hemorrhage (I61), or subarachnoid hemorrhage (I60)]. Medical tests used to identify ischemic heart disease and heart failure were electrocardiogram and echocardiograph. Medical tests used to identify stroke were computed tomography and magnetic resonance imaging.

### Statistical analysis

Incidence rates in each cohort were calculated as the number of cases per total period at risk for CVD. Crude and age–sex adjusted incidence rate ratios (IRRs) with 95 % confidence intervals (CIs) versus OA were also calculated on the assumption of a Poisson distribution.

Univariate and multivariate Cox-regression analyses were performed, including hazard ratios (HRs), 95 % CIs, and Wald test *P* values to evaluate risk factors for CVD in RA patients. The candidate covariates were age, sex, CRP at baseline, total cholesterol at baseline, and diabetes complications. CRP was selected as an index of systemic inflammation associated with RA. Total cholesterol and diabetes complications were selected as traditional risk factors for CVD. CRP and total cholesterol at baseline were measured on the date of or before first diagnosis of RA or OA. No variable selection techniques were used in multivariate Cox-regression analyses. Statistical analyses were performed using SAS version 9.3.

### Ethics approval

Our study was approved by the institutional review board of the Public Health Research Foundation (http://www.phrf.jp/) on 5 November 2014 and was conducted according to the local ethical guidelines for epidemiological research. Informed consent was waived by the institutional review board because the database used in this study is anonymously managed and includes no personal information.

## Results

A total of 8658 patients with RA (the RA cohort) and 32,202 patients with OA were identified. Laboratory data for analyzing risk factors were available for 2472 patients with RA (the RA sub-cohort). Patients with RA were younger and less likely to have diabetes complications than patients with OA (Table 1). Sex distribution and total cholesterol at baseline were similar in both groups.

**Table 1:** Characteristics of the study populations

Characteristics	RA patients		OA patients
	All cohort	Sub-cohort^a^	
Number of patients, n	8658	2472	32,202
Female, n (%)	5819 (67.2)	1711 (69.2)	22,046 (68.5)
Age, mean (SD) (years)	63.8 (15.1)	62.4 (15.3)	70.3 (12.0)
Median (years)	66.0	64.0	72.0
Diabetes complications, n (%)	424 (4.9)	90 (3.6)	2571 (8.0)
CRP at baseline, mean (SD) (mg/dl)	–	2.4 (4.1)	–
Median (mg/dl)	–	0.7	–
Total cholesterol at baseline, mean (SD) (mg/dl)	–	186.9 (39.9)	–
Median (mg/dl)	–	183.0	–

*RA* rheumatoid arthritis, *OA* osteoarthritis, *SD* standard deviation, *CRP* C-reactive protein

^a^Patients with laboratory data who were included in risk factor analysis

In the RA cohort (n = 8658), the age-sex adjusted IRRs for RA versus OA were statistically high for total CVD (2.12; 95 % CI 1.93–2.32), ischemic heart disease (2.16; 95 % CI 1.86–2.50), myocardial infarction (3.62; 95 % CI 2.52–5.18), heart failure (2.34; 95 % CI 2.07–2.65), and stroke (1.68; 95 % CI 1.41–2.00) (Table 2).

**Table 2:** Incidence rate ratios of cardiovascular disease

	RA patients (N = 8658)		OA patients (N = 32,202)		RA versus OA	
	Events	PYs at risk	Events	PYs at risk	Crude IRR (95 % CI)	Adjusted IRR^a^ (95 % CI)
CVD (total)	673	10,946	1633	43,304	1.63 (1.49–1.78)	2.12 (1.93–2.32)
Ischemic heart disease	260	11,280	586	44,091	1.73 (1.50–2.01)	2.16 (1.86–2.50)
Myocardial infarction	53	11,481	71	44,574	2.90 (2.03–4.14)	3.62 (2.52–5.18)
Angina pectoris	224	11,319	528	44,138	1.65 (1.41–1.93)	2.05 (1.75–2.40)
Heart failure	358	11,234	808	44,003	1.74 (1.53–1.97)	2.34 (2.07–2.65)
Stroke	177	11,396	513	44,286	1.34 (1.13–1.59)	1.68 (1.41–2.00)
Cerebral infarction	136	11,433	431	44,327	1.22 (1.01–1.48)	1.56 (1.29–1.90)
Intracerebral hemorrhage	32	11,506	77	44,599	1.61 (1.07–2.43)	1.85 (1.22–2.81)
Subarachnoid hemorrhage	15	11,517	15	44,626	3.87 (1.89–7.93)	4.45 (2.13–9.26)

*RA* rheumatoid arthritis, *OA* osteoarthritis, *PYs* patient-years, *IRR* incidence rate ratio, *CI* confidence interval, *CVD* cardiovascular disease

^a^Adjusted by age and sex

In univariate analysis of the RA sub-cohort (n = 2472), HRs showed that higher age (8.50; 95 % CI 5.13–14.09) and higher CRP (2.00; 95 % CI 1.39–2.88) at baseline significantly increased the risk for CVD, but no statistical significance was observed for sex, total cholesterol at baseline, and diabetes complications (Table 3). In multivariate analysis, we excluded total cholesterol at baseline from the covariates because approximately half of patients had no baseline total cholesterol data. Adjusted HRs based on the multivariate model presented in Table 3 showed that higher age is an independent risk factor for CVD (HR 8.52; 95 % CI 4.76–15.23). Higher baseline CRP showed a trend of increasing CVD risk, although the difference was not statistically significant (HR 1.43; 95 % CI 0.99–2.07). No significant differences were observed for sex or diabetes complications. The median baseline CRP level in patients with CVD events (1.6 mg/dl) was higher than in patients without CVD events (0.7 mg/dl). In addition, the median CRP level at CVD onset (1.7 mg/dl) had not decreased from baseline.

**Table 3:** Risk factors for cardiovascular disease in RA patients estimated by univariate and multivariate Cox proportional hazard regression model

	Univariate model		Multivariate model	
	Hazard ratio (95 % CI)	*P* value	Hazard ratio (95 % CI)	*P* value
Male	1.07 (0.76–1.51)	0.6812	1.02 (0.70–1.49)	0.8990
Age ≥65 years	8.50 (5.13–14.09)	<0.0001	8.52 (4.76–15.23)	<0.0001
CRP at baseline ≥1.0 mg/dl	2.00 (1.39–2.88)	0.0002	1.43 (0.99–2.07)	0.0564
Total cholesterol at baseline ≥220 mg/dl^a^	0.68 (0.34–1.38)	0.2883	–	–
Diabetes complications	1.86 (0.98–3.54)	0.0577	1.72 (0.83–3.56)	0.1436

*RA* rheumatoid arthritis, *CRP* C-reactive protein

^a^Total cholesterol at baseline was excluded from covariates in the multivariate model because there was no significant association between total cholesterol and CVD as well as a high proportion of missing data

## Discussion

CVD can be a fatal complication in RA patients, and understanding CVD risk in Japanese RA patients is important for risk management. High prevalence of cardiovascular comorbidities in patients with RA in Japan was recently shown, using a database of medical claims from employment-based health insurance organizations (Sakai et al. 2016). However, this database covers only company employees and their dependents, who are considered to be younger and at lower risk for CVD than the retired population. We used a database that includes data on patients of all ages from hospitals located throughout Japan.

Our study shows increased risk for CVD in RA patients. Adjusted IRRs for RA versus OA were 2.12 (95 % CI 1.93–2.32) for total CVD, 2.16 (95 % CI 1.86–2.50) for ischemic heart disease, 3.62 (95 % CI 2.52–5.18) for myocardial infarction, and 1.68 (95 % CI 1.41–2.00) for stroke. The IRR for ischemic heart disease in this study was similar to the odds ratio (RA vs. non-RA population) for ischemic heart disease recently reported from another study in Japan (Sakai et al. 2016), and the IRRs for myocardial infarction and stroke in this study were similar to the odds ratios (RA vs. non-RA population) for myocardial infarction and stroke observed in a US study (Solomon et al. 2006).

Our results are the first to show an association between systemic inflammation and CVD in Japanese patients with RA. The risk factor analyses identified advanced age as a risk factor for CVD in RA patients. The results also suggest that higher baseline CRP increases CVD risk whereas higher total cholesterol at baseline, one of the traditional risk factors for CVD, does not. The baseline CRP level in patients with CVD events was higher than in patients without CVD events, and CRP level at CVD onset had not decreased from baseline. These results imply an association between systemic inflammation in RA and risk for CVD, which is consistent with previous reports (Goodson et al. 2005). Active treatment to reduce systemic inflammation may be one way to reduce CVD risk.

It is important to note the limitations of this study. First, the database for this study only covers some of the acute care hospitals in Japan. Therefore, the results in this study might not represent all RA patients in Japan. Second, we could not follow patients over the long term. The median follow-up period for RA patients in the database was 438 days, which is shorter than in other studies. In spite of this short follow-up period, we observed a higher risk for CVD in RA patients than in OA patients. Third, information could not be obtained from transferring or recipient hospitals. Therefore, outcome information might be missing for some patients. Last, the database used does not include some of the essential patient background data for traditional CVD risk factors such as history of CVD, body mass index, blood pressure, and smoking history, so we could not exclude from analysis the confounding bias of these factors. Despite these limitations, this large-scale database study presents valuable information to help characterize the risk for CVD in Japanese patients with RA.

## Conclusion

In conclusion, our study indicates an increased risk for CVD and an association between systemic inflammation and CVD in patients with RA in Japan. Further epidemiological investigations in Japan could be expected to support these results.
